# Functional analysis of the acetylation of human p53 in DNA damage responses

**DOI:** 10.1007/s13238-014-0048-x

**Published:** 2014-04-02

**Authors:** Sun-Ku Chung, Shengyun Zhu, Yang Xu, Xuemei Fu

**Affiliations:** 1 Shenzhen Children’s Hospital, 7019 Yitian Road, Shenzhen, Guangdong 518026 China; 2 Division of Biological Sciences, University of California, San Diego, 9500 Gilman Drive, La Jolla, CA 92093 USA; 3 Chongqing Medical University, Chongqing, 400016 China

**Keywords:** human embryonic stem cells (hESCs), p53, acetylation, homologous recombination, DNA damage, cancer

## Abstract

As a critical tumor suppressor, p53 is inactivated in human cancer cells by somatic gene mutation or disruption of pathways required for its activation. Therefore, it is critical to elucidate the mechanism underlying p53 activation after genotoxic and cellular stresses. Accumulating evidence has indicated the importance of posttranslational modifications such as acetylation in regulating p53 stability and activity. However, the physiological roles of the eight identified acetylation events in regulating p53 responses remain to be fully understood. By employing homologous recombination, we introduced various combinations of missense mutations (lysine to arginine) into eight acetylation sites of the endogenous p53 gene in human embryonic stem cells (hESCs). By determining the p53 responses to DNA damage in the p53 knock-in mutant hESCs and their derivatives, we demonstrate physiological importance of the acetylation events within the core domain (K120 and K164) and at the C-terminus (K370/372/373/381/382/386) in regulating human p53 responses to DNA damage.

## Introduction

Tumor suppressor p53 is a transcription factor that directly activates the transcription of hundreds of genes, including p21, Mdm-2, noxa, and puma that are mediating p53-dependent functions (Vousden and Prives, 2009). In addition, p53 also directly suppresses the expression of a number of genes, such as Nanog (Lin et al., 2005). While p53 remains in an unstable and inactive state in normal cells in the absence of exogenous stresses, its protein level and transcription activity are significantly induced in response to DNA damage and other cellular stresses (Vousden and Prives, 2009). The interaction between p53 and E3 ligases such as MDM2, all transcription targets of p53, leads to the ubiquitination and rapid degradation of p53. In addition, the interaction between p53 and Mdm2 or MdmX suppresses the transcriptional activities of p53. These negative regulatory pathways are critical to prevent the hyperactivation of p53 that can have devastating effects on organismal survival and aging (Campisi, 2005; Liu et al., 2010).

p53 is critical to suppress cancer development in humans, and the loss of wild-type p53 activity through direct somatic gene mutation or disruption of pathways important for p53 activation is required for cancer progression (Vousden and Prives, 2009). Therefore, it is critical to elucidate the pathways that activate p53. Accumulating evidence indicates that posttranslational modifications of p53, including phosphorylation and acetylation, are involved in regulating p53 stability and activity. While phosphorylation of p53 at N-terminus is important to activate some of p53-dependent functions, none of the phosphorylation events are critical for p53 activation (Chao et al., 2003; Chao et al., 2006). Recent studies indicate that human p53 can be acetylated at multiple lysine residues by Tip60 and CBP, including K120, K164, K320, and the C-terminal K370/372/373/381/382/386 (Brooks and Gu, 2011). Using human cancer cell lines expressing p53 with various lysine to arginine mutations, these studies indicate that K120R, K164R as well as K120/164R double mutations partially impaired the p53-dependent functions (Sykes et al., 2006; Tang et al., 2006; Tang et al., 2008). In addition, mutation of the eight acetylation sites to arginine abolishes the p53-dependent functions, suggesting that these acetylation events play synergistic and critical roles in activating p53 (Sykes et al., 2006; Tang et al., 2006, 2008).

The roles of acetylation have also been examined by knock-in studies in mouse and ESCs. Employing p53 K3R knock-in mouse, Li and colleagues demonstrate that acetylation of mouse p53 at K117/161/162 (corresponding to human K120/164) is required for activating p53-dependent transcription but dispensable for p53 stabilization (Li et al., 2012). By generating p53 K120/164R knock-in hESCs, we demonstrate that these two acetylation events are required for p53-dependent transcription and stabilization (Zhang et al., 2014). These findings demonstrate that acetylation might have distinct impact on p53 stability and activity in mouse and human cells.

## Results

### Generation of K6R and K8R knock-in hESCs

**Figure 1:**
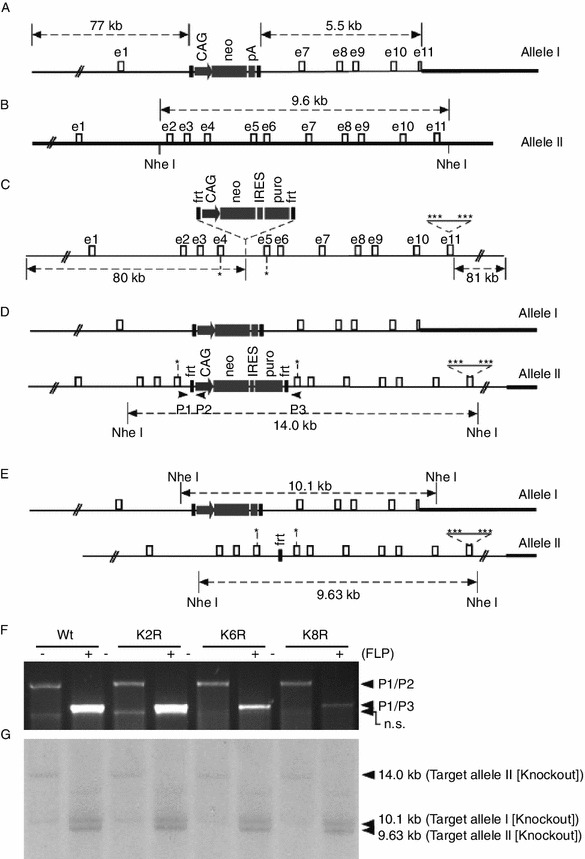
Generation of p53 acetylation site knock-in hESCs. (A) Genome configuration of the p53 knockout allele in parental p53^+/−^ hESCs. (B) Germline configuration of p53 WT allele in p53^+/−^ hESCs. (C) The BAC-based knock-in vector to introduce K to R mutations into the endogenous p53 gene in hESCs. The eight K to R mutations are indicated by asterisks. (D) Homologous recombination between the WT p53 allele of p53^+/−^ hESCs and the targeting vector led to knock-in hESCs with the selection cassette inserted in the targeted allele. The PCR primers to screen for the FLP/FRT-mediated deletion are indicated. (E) Knock-in hESCs. Transient expression of FLP in the targeted hESCs led to the excision of the selection marker from the knock-in allele. The sizes of the NheI restriction fragments are indicated. (F) PCR (top panel) and Southern blotting (bottom panel) analyses of the knock-in hESCs. Deletion of the selection cassette led to the loss of PCR amplification by primers P1/P2 and the gain of PCR amplification by primers P1/P3. Genomic DNA was digested by Nhe1 for Southern blotting

**Figure 2:**
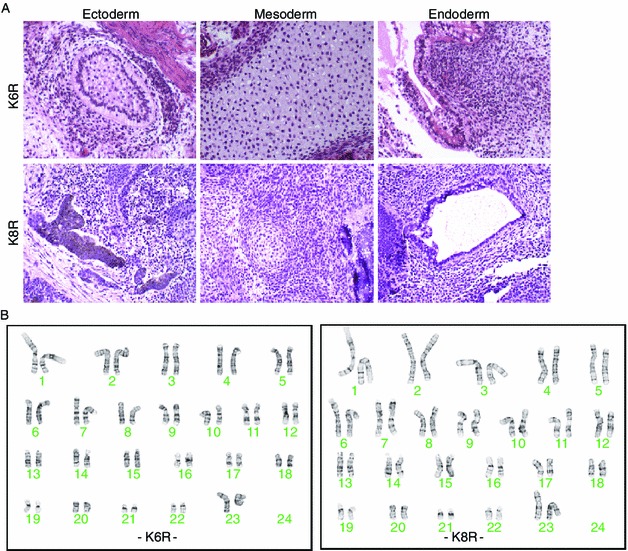
Characterization of p53 K6R and K8R knock-in hESCs. (A) Metaphase cells of the knock-in hESCs were examined and exhibited normal karyotypes. Representative images are shown. (B) Knock-in hESCs formed well-differentiated teratomas in SCID mice. Cells of each of the three germ layers were identified in the teratomas

To determine the functional interaction of the acetylation events within the core domain and the C-terminus of p53, we employed the same genetic approach to reveal the physiological importance of eight acetylation events in regulating human p53. The strategy to introduce the K to R mutations (K370/372/373/381/382/386R, K6R; K120/164/370/372/373/381/382/386R, K8R) into the endogenous p53 gene of hESCs is the same as we described for the K120/164R (K2R) knock-in mutation (Zhang et al., 2014) and is described in Fig. 1. We introduced K8R mutations into two independent hESC lines (HUES8 and HUES9) and K6R mutations into the HUES8 hESCs. These knock-in hESCs have normal karyotypes and can form well-differentiated teratomas in immunodeficient mice, confirming their pluripotency (Fig. 2A and 2B). The full length cDNA of the p53 gene in the knock-in hESCs was sequenced to confirm that only the K to R mutations but no other mutations were introduced into the p53 knock-in allele.

### p53 stabilization is abolished in K8R hESCs and fibroblasts after DNA damage

Our previous studies have shown that K120/164R (K2R) mutation is important for p53 stabilization and activation in hESCs after DNA damage (Zhang et al., 2014). Cell lines studies indicate that the acetylation at K120/164 works synergistically with acetylation at the C-terminus (Tang et al., 2008). To determine the physiological roles of the acetylation of p53, we examined the impact of K8R mutations on the p53 stability and activity in hESCs after DNA damage. Consistent data were obtained from both K8R knock-in hESC lines (HUES8 and HUES9), and the representative data are presented.

**Figure 3:**
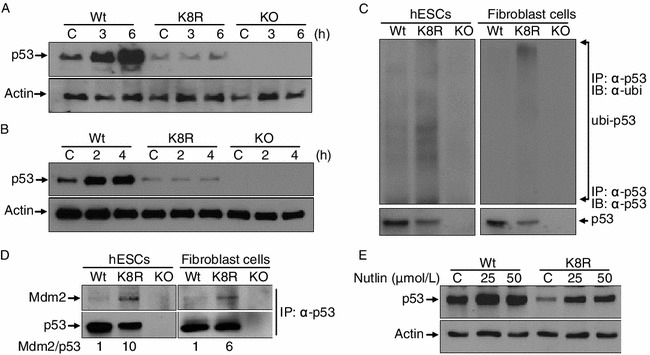
p53 stabilization is compromised in K8R hESCs and fibroblasts. The protein levels of p53 in p53^+/−^, K8R and p53^−/−^ hESCs (A) and fibroblasts (B) at different time points after DNA damage induced by doxorubicin. The genotypes and the hours of doxorubicin treatment are indicated. The basal levels of p53 in K8R cells are lower than those of p53^+/−^ cells. (C) The ubiquitination of p53 in p53^+/−^ and K8R hESCs and fibroblasts. p53^−/−^ hESCs and fibroblasts were used as negative controls for specificity of immunoprecipitation. (D) The interaction between p53 and Mdm2 in p53^+/−^ and K8R cells by co-immunoprecipitation. p53 was immunoprecipitated from p53^+/−^ and K8R cells, and the levels of p53 and Mdm2 in the immunoprecipitate were determined by Western blotting. (E) The stabilization of p53 in p53^+/−^ and K8R hESCs by treating with Nutlin-3 for 24 h. The concentrations of Nutlin-3 used are indicated on the top

To determine the impact of K8R on p53 stability, the protein levels of p53 in p53^+/−^, K8R and p53^−/−^ hESCs at different time points after DNA damage. While the protein levels of p53 were significantly increased in p53^+/−^ hESCs after DNA damage, they remained unchanged in K8R hESCs after DNA damage, indicating that DNA damage induced stabilization of p53 is abolished in K8R hESCs (Fig. 3A). Consistent with the findings that p53 is constitutively acetylated (Tang et al., 2008), the basal levels of p53 protein were significantly lower in K8R hESCs than in p53^+/−^ hESCs, indicating that acetylation stabilizes p53 in the absence of exogenous stresses.

To confirm that the role of this acetylation in stabilizing p53 is not cell-type specific, we derived the fibroblasts from p53^+/−^ and K8R hESCs, and determined the p53 protein levels in these cells after DNA damage. Our data confirmed that the basal levels of p53 protein were much lower in K8R fibroblasts than those in p53^+/−^ fibroblasts and could not be induced by DNA damage (Fig. 3B). Therefore, we concluded that acetylation is required to stabilize p53. In further support of this conclusion, p53 was hyper-ubiquitinated in K8R hESCs and fibroblasts when compared with that in p53^+/−^ cells (Fig. 3C). Consistent with the increased ubiquitination of p53 in K8R cells, the interaction between Mdm2 and p53 was significantly increased in K8R hESCs than in p53^+/−^ hESCs when analyzed by co-immunoprecipitation (Fig. 3D). In addition, treatment with Nutlin-3, which specifically disrupts the interaction between Mdm2 and p53, significantly increases the protein levels of p53 in K8R hESCs, supporting the notion that the decreased protein levels of p53 in K8R hESCs are primarily due to the increased p53-Mdm2 interaction (Fig. 3E). In summary, these findings support the notion that acetylation is required for p53 stabilization by promoting p53-Mdm2 interaction.

### p53-dependent functions are abolished in K8R hESCs and fibroblasts

**Figure 4:**
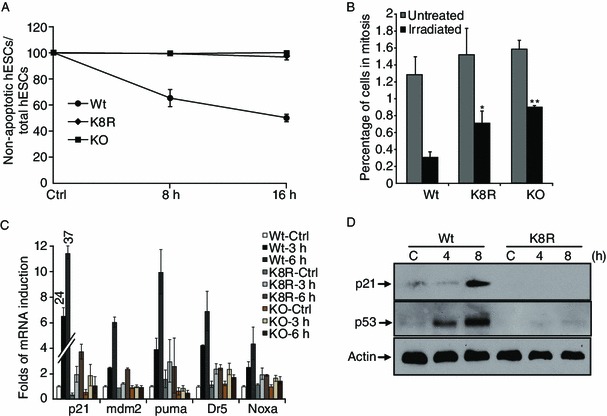
p53-dependent functions are abolished in K8R hESCs and functions. (A) p53-dependent apoptosis in p53^+/−^, K8R and p53^−/−^ hESCs after 5 Gy of IR. The apoptotic cells were identified by staining with Annexin V. (B) The p53-dependent G_2_/M checkpoint in p53^+/−^, K8R and p53^−/−^ hESCs 1 h after 5 Gy of IR. Mean values from three independent experiments are shown with SD. The *P* value between K8R cells and p53^+/−^ cells is 0.02 (*), while the *P* value between p53^−/−^ cells and p53^+/−^ cells is 0.001 (**) as shown is Fig. 4B. (C) p53-dependent gene expression in p53^+/−^, K8R and p53^−/−^ hESCs after doxorubicin treatment. The mRNA levels of p53 target genes were determined by quantitative real time PCR and standardized by the mRNA levels of GAPDH. (D) p53-dependent induction of p21 is abolished in the fibroblasts derived from K8R hESCs after doxorubicin treatment. The time points after the treatment are indicated on the top

Previous findings have shown that hESCs undergo p53-dependent apoptosis and cell cycle G_2_/M checkpoint after DNA damage (Song et al., 2010). Therefore, these p53-dependent functions were analyzed in K8R hESCs after DNA damage. p53-dependent apoptosis was abolished in the K8R hESCs after DNA damage induced by doxorubicin (Fig. 4A). In addition, p53-dependent G_2_/M checkpoint was essentially compromised in K8R hESCs after IR (Fig. 4B). Consistent with these findings, p53-dependent transcription of target genes, including p21, Mdm2, and Puma, was abolished in K8R hESCs after IR (Fig. 4C). To confirm that the impact of K8R mutation on p53 transcription activity is not cell type dependent, we analyzed the p53-dependent induction of p21 in the fibroblasts derived from K8R hESCs, indicating that p53-dependent p21 expression is abolished in K8R fibroblasts both before and after DNA damage (Fig. 4D). Therefore, we concluded that p53-dependent functions are abolished in K8R hESCs after DNA damage.

### Functional dissection of acetylation within the core domain and C-terminus

**Figure 5:**
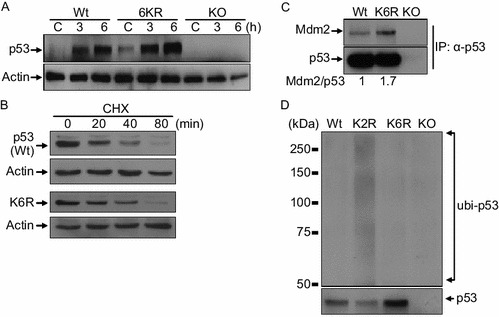
The stability of p53 protein in K6R knock-in hESCs. (A) The protein levels of p53 in p53^+/−^ and K6R hESCs after doxorubicin treatment. The p53 and actin are indicated. (B) The protein levels of p53 in p53^+/−^ and K6R hESCs at different time points after treatment with cycloheximide (CHX) that inhibits protein synthesis. (C) The interaction between p53 and Mdm2 in p53^+/−^ and K6R hESCs was analyzed by co-immunoprecipitation. p53^−/−^ hESCs were used as a negative control. (D) The ubiquitination of p53 in p53^+/−^, K2R and K6R hESCs. The p53 protein was immunoprecipitated and the amount of ubiquitination was analyzed by anti-ubiquitin antibody. p53^−/−^ hESCs were used as a negative control

To dissect the contribution of the acetylation events within the core domain and C-terminus to activate p53 responses, we examined the p53 responses to DNA damage in K6R knock-in hESCs and compared to those in K2R (K120/164R) hESCs. In contrast to the findings that p53 protein levels in K8R hESCs were much lower than those in p53^+/−^ hESCs and could not be increased by DNA damage, the p53 protein levels in K6R hESCs were induced by DNA damage (Fig. 5A). While the half-life of p53 in K2R hESCs is significantly reduced when compared to that in p53^+/−^ hESCs (Zhang et al., 2014), the half-life of p53 in p53^+/−^ hESCs is similar to that in K6R hESCs (Fig. 5B). In contrast to that of K8R, the interaction between p53 and Mdm2 is only modestly increased in K6R hESCs, indicating that acetylation within the core domain plays a key role in disrupting the interaction between p53 and Mdm2 (Fig. 5C). However, the ubiquitination levels were modestly reduced in K6R hESCs, supporting the notion that the C-terminal lysine residues are targeted for ubiquitination (Fig. 5D).

### The p53-dependent functions in K6R hESCs and fibroblasts

**Figure 6:**
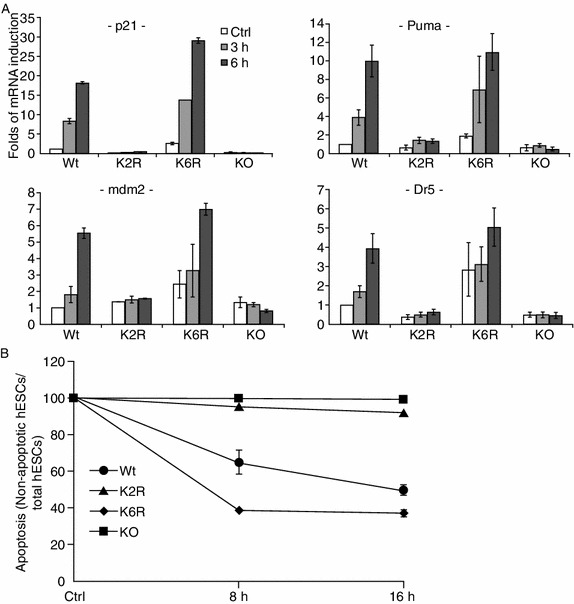
p53-dependent functions are abolished in K2R cells but increased in K6R cells. (A) p53-dependent gene expression in p53^+/−^, K2R and K6R hESCs after doxorubicin treatment (0.2 μmol/L). The mRNA levels of each p53 target genes were determined by quantitative PCR and standardized with the mRNA levels of GAPDH. (B) p53-dependent apoptosis in p53^+/−^, K2R and K6R hESCs after doxorubicin treatment. The apoptotic cells were identified by staining with Annexin V. Mean value from three independent experiments are shown with SD. *n* = 3

In contrast to that in K8R and K2R hESCs that are abolished in p53-dependent gene expression after DNA damage (Fig. 6A), p53-dependent gene expression was higher in K6R hESCs than those in p53^+/−^ control hESCs both before and after DNA damage, indicating that K6R mutation increases the p53 responses to DNA damage in hESCs (Fig. 6A). In further support of this conclusion, p53-dependent apoptosis was abolished in K2R hESCs but increased in K6R hESCs after IR (Fig. 6B). Therefore, in contrast to the cell line data suggesting that K2R and K6R mutations synergistically disrupt p53-dependent transcription but not p53 stability (Li et al., 2012), our findings indicate that K2R and K6R mutations have opposite impacts on p53 stability and activity.

## Discussion

By generating acetylation site mutant knock-in hESCs, we revealed the physiological roles of acetylation events within the core domain and C-terminus and their functional interaction in activating human p53. There are several distinctions between our conclusion and those reached in previous cell line and mouse studies (Tang et al., 2008; Li et al., 2012). First, while previous findings indicate that acetylation is required for p53-dependent activity but dispensable for p53 stabilization, our findings indicate that acetylation is required for p53 stabilization. Second, previous findings suggest that the acetylation at the core domain plays synergistic roles with the acetylation at the C-terminus in activating p53-dependent transcription. However, our findings indicate that the mutations of the acetylation sites within the core domain and at the C-terminus have opposite impact on p53 stability and activity. These discrepancies could be due to the intrinsic difference in the sequence of the mouse and human p53 gene. In addition, the overexpression of various p53 mutants in human cancer cell lines could also mask the important roles of acetylation in p53 stabilization.

Our findings indicate that acetylation within the core domain but not at the C-terminus plays a key role in the disruption of the interaction between p53 and its E3 ligase Mdm2. In this context, in addition to the N-terminus of p53, Mdm2 binds to other regions of p53, including the core domain (Vousden and Prives, 2009). Despite the finding that p53-Mdm2 interaction is modestly increased in K6R cells, p53 is more stable in K6R cells with reduced ubiquitination when compared to wild type p53, indicating that the C-terminal lysine residues are the preferential targets for ubiquitination. In addition, the increased stability of p53 in K6R cells after DNA damage leads to higher p53-dependent transcription and apoptosis after DNA damage. Together with previous findings, our data demonstrate the critical and complex roles of acetylation in activating human p53 response to DNA damage and could provide the foundation to develop novel strategies to activate p53 in human cancer cells harboring WT but dysfunctional p53.

## Materials and methods

### Generation of acetyl defective p53 mutants

RP11-199F11, BAC clone including human p53, was purchased from Invitrogen. The BAC based targeting vector was generated and modified by recombineering in the *E. coli* strain SW102. To obtain p53^+/−^ hESCs, Neomycin marker was firstly used for selection (Song et al., 2010). Each of wild type, or mutant p53 BAC vector commonly had selection cassette (CAG-Neo-IRES-Puro-polyA) through insertion into intron 4 of p53. By targeting each BAC DNA into another allele of p53^+/−^ hESCs through homologous recombination, each clone harboring wild type, or acetyl mutant of p53 was obtained from p53^+/−^ hESCs. After transfecting FLP gene into each clone, cDNA sequence of each clone was confirmed to mutation sites.

### Cell culture for hESCs

In the presence of feeder layer, the HUES cells were cultured on feeder layer in knockout DMEM supplemented with 10% knockout serum replacement (KSR), 10% plasmanate, 1% PenStrep, 1% Glutamine, 1% nonessential amino acids, 10 ng/mL bFGF, and 55 μmol/L β-mercaptoethanol as described (Cowan et al., 2004). In the absence of feeder layer, HUES cells were cultured on matrigel-coated plates in the mTeSR1 medium with 5× supplement. All tissue culture reagents were purchased from Invitrogen.

### Western blotting and immunoprecipitation analysis

Protein extracts from hESCs or fibroblasts derived from teratoma were loaded on 6%–10% SDS-PAGE gel and transferred to nitrocellulose membrane, which was probed with a monoclonal antibody against p53 (pAb1801; Santa Cruz Biotechnology, Santa Cruz, CA), monoclonal antibody against Mdm2 (Ab-2; Oncogene Research), polyclonal antibody against p21 or β-actin (Santa Cruz Biotechnology). The membrane was subsequently probed with a horseradish peroxidase-conjugated secondary antibody and developed with ECL PLUS (Amersham, Piscataway, NJ). For co-immunoprecipitation analysis, 1–2 mg of whole cell protein extracts was immunoprecipitated with anti-p53 antibody (FL393; Santa Cruz Biotechnology). The amount of p53 and Mdm2 in the immunoprecipitate was analyzed by Western blotting using monoclonal antibody against p53 (pAb1801; Santa Cruz Biotechnology, Santa Cruz, CA), monoclonal antibody against Mdm2 (SMP-14; Santa Cruz Biotechnology, Santa Cruz, CA).

### Quantitative real-time PCR

Real-time PCR was performed as previously described (Song et al., 2010). The sequence of the primers was as follows: p21, 5′-ACCTGGAGACTCTCAGGGTCG-3′ for forward primer, 5′-TTAGGGCTTCCTCTTGGAGAAGAT-3′ for reverse primer; Mdm2, 5′-ATCGGACTCAGGTACATCTGTGAG-3′ for forward primer, 5′-AGGTTTCTCTTCCTGAAGCTCTTG-3′ for reverse primer; Puma, 5′-GACGACCTCAACGCACAG-3′ for forward primer, 5′-CTAATTGGGCTCCATCTCG-3′ for reverse primer; Noxa, 5′-TCCAGTTGGAGGCTGAGGTT-3′ for forward primer, 5′-CACTCGACTTCCAGCTCTGC-3′ for reverse primer; Killer5, 5′-CTCCTGCAAATATGGACAGGACTA-3′ for forward primer, 5′-TTAGCTCCACTTCACCTGAATCAC-3′ for reverse primer.

### Apoptosis assays

hESCs were cultured onto matrigel-coated plates in mTeSR1 medium with 5× supplement (Invitrogen). hESCs were treated with doxorubicin (0.2 μmol/L), or irradiated with γ-irradiation (5 Gy), and harvested 8 and 16 h after treatment. Apoptotic cells were identified by staining with Annexin V as previously described (Chao et al., 2003).

### *In vivo* ubiquitylation assays

For *in vivo* ubiquitylation assays, prior to cell collection, cells were treated with Alln (25 μmol/L), or MG132 (25 μmol/L) for 4 h, and then cells were lysed in modified RIPA buffer (1% Nonidet P-40, 0.1% sodium dodecyl sulfate [SDS], Tris-HCl [pH 7.8], 150 mmol/L NaCl, 1 mmol/L dithiothreitol, 0.5 mmol/L EDTA, 25 μmol/L Alln, 25 μmol/L MG132, 5 mmol/L *N*-ethylmaleimide, and fresh proteinase inhibitors) with mild sonication. Cell lysates were immunoprecipitated with anti-p53 antibody (FL393 AC; Santa Cruz Biotechnology, Santa Cruz, CA) and analysed by Western blotting using anti-ubiquitin antibody (P4D1; Santa Cruz Biotechnology, Santa Cruz, CA) or anti-p53 antibody (pAb1801; Santa Cruz Biotechnology) (Feng et al., 2005).

### Analysis of p53 stability

For *in vivo* p53 degradation assays, hESCs were incubated with cycloheximide (100 μg/mL). Cells were harvested at 20, 40, and 80 min later, subjected to Western blotting using anti-p53 antibody (pAb1801; Santa Cruz Biotechnology, Santa Cruz, CA). The intensity of bands was measured by densitometry.

### Karyotyping analysis

Karyotyping of the mutant hESC clones was carried out by Cell Line Genetics (Madison, WI).
